# 德曲妥珠单抗治疗获得性*HER2*高水平扩增的*EGFR*突变肺腺癌：病例报道

**DOI:** 10.3779/j.issn.1009-3419.2026.104.01

**Published:** 2026-03-20

**Authors:** Xiaoxiao GE, Hefeng CHEN, Shaohua CUI

**Affiliations:** ^1^200040 上海，复旦大学附属华东医院呼吸与危重症医学科; ^1^Department of Pulmonary and Critical Care Medicine, Huadong Hospital, Fudan University, Shanghai 200040, China

**Keywords:** 肺肿瘤, 德曲妥珠单抗, HER2扩增, 获得性耐药, 病例报道, Lung neoplasms, Trastuzumab deruxtecan, HER2 amplification, Acquired resistance, Case report

## Abstract

德曲妥珠单抗是新近研发的抗体偶联药物（antibody-drug conjugate, ADC），用于克服人生长因子受体2（human epidermal growth factor receptor 2, HER2）异常改变。研究已证实其用于既往接受过治疗的HER2突变型非小细胞肺癌（non-small cell lung cancer, NSCLC）患者，疗效可观且安全性可控。然而，该药用于治疗获得性HER2扩增的NSCLC的疗效数据较为有限。本文报道1例转移性肺腺癌患者，在二线奥希替尼治疗后出现获得性HER2的高水平扩增（扩增倍数达20拷贝），经减量德曲妥珠单抗治疗后达到部分缓解（partial response, PR）。血浆二代测序（next-generation sequencing, NGS）结果进一步证实HER2扩增被成功抑制。

## Abstract (en)

Trastuzumab deruxtecan is a newly developed antibody-drug conjugate (ADC) designed to overcome human epidermal growth factor receptor 2 (HER2) aberrations. Studies have confirmed that Trastuzumab deruxtecan yields promising efficacy with a manageable safety profile in previously treated non-small cell lung cancer (NSCLC) patients harboring HER2 mutations. However, data on efficacy of Trastuzumab deruxtecan for acquired HER2-amplified NSCLC are limited. Here, we report a case of a patient with metastatic lung adenocarcinoma who developed acquired high-level HER2 amplification (20 copies) after 2^nd^-line Osimertinib. A partial response (PR) was achieved with reduced-dose Trastuzumab deruxtecan. Plasma next-generation sequencing (NGS) further confirmed successful inhibition of HER2 amplification.

人表皮生长因子受体 2（human epidermal growth factor receptor 2, HER2），亦称Erb-B2受体酪氨酸激酶2（Erb-B2 receptor tyrosine kinase 2, ERBB2），是一种已在包括非小细胞肺癌（non-small cell lung cancer, NSCLC）在内的多种肿瘤中得到验证的可靶向生物标志物^[1]^。德曲妥珠单抗（DS-8201a, Trastuzumab deruxtecan, T-DXd, Enhertu）是新近研发的抗体偶联药物（antibody-drug conjugate, ADC），用于克服HER2异常改变。DESTINY-Lung01^[2]^与DESTINY-Lung02^[3]^研究已证实，德曲妥珠单抗用于既往接受过治疗的HER2突变型NSCLC患者，疗效可观且安全性可控^[2,3]^。然而，HER2异常改变除基因突变外，还包括基因扩增与蛋白过表达^[4]^。研究^[5]^发现，肺癌中HER2基因扩增与基因突变属于不同类型的作用靶点。此外，NSCLC中的HER2扩增既可以作为原发异常出现在未经治疗的患者中，也可作为继发或获得性异常发生于接受表皮生长因子受体-酪氨酸激酶抑制剂（epidermal growth factor receptor-tyrosine kinase inhibitors, EGFR-TKIs）治疗后的患者^[5-8]^。目前，德曲妥珠单抗用于HER2扩增型NSCLC的疗效数据仍较为有限。本文报道1例接受德曲妥珠单抗治疗有效的获得性HER2扩增转移性肺腺癌病例。本病例报告遵循病例报告指南（CAse REport guidelines, CARE）撰写。

## 1 病例资料

患者女，73岁，自2018年2月起出现反复咳嗽（图1）。既往存在亚临床甲状腺功能减退症，无吸烟史及家族肿瘤史。体格检查提示美国东部肿瘤协作组体力状态（Eastern Cooperative Oncology Group performance status, ECOG PS）评分为1分，余未见明显异常。胸部增强计算机断层扫描（computed tomography, CT）提示右肺上叶存在软组织肿块。排除远处转移后，行胸腔镜下右肺上叶切除术加纵隔淋巴结清扫术。术后病理检查确诊为多原发肺腺癌，病理分期分别为IA和IB期。分子病理检测提示EGFR 18号外显子G719X突变。术后给予4个周期培美曲塞联合顺铂方案辅助化疗。随后患者接受定期随访。2021年10月，复查胸部CT提示双肺、纵隔及胸椎多发转移，考虑疾病复发。鉴于患者存在EGFR G719X突变，予以阿法替尼一线治疗（40 mg口服，每日1次）。2021年12月，根据实体瘤疗效评价标准1.1（Response Evaluation Criteria in Solid Tumors 1.1, RECIST 1.1）评估疗效为部分缓解（partial response, PR），患者继续阿法替尼治疗直至2023年2月出现全身进展（累及脑、肺、骨及肾上腺）。对进展的右肺病灶再次行活检，病理示中分化腺癌，分子病理检测提示EGFR L858R突变。随后，予奥希替尼二线治疗（80 mg口服，每日1次）。尽管患者对奥希替尼初始治疗有效，但6个月后出现肝转移及肾上腺病灶进展。考虑到肿瘤进展缓慢且患者仍可从药物中获益，遂继续予以奥希替尼，同时对肾上腺病灶行局部放疗。然而3个月后（2023年11月），患者出现全身淋巴结转移，因此停用奥希替尼。此时，患者ECOG PS评分为2分，予以培美曲塞单药三线治疗，但给药1次后患者即出现4级骨髓抑制。同时，我们对患者左侧颈部肿大淋巴结行活检，提示为肺腺癌转移。对标本行下一代测序（next-generation sequencing, NGS），结果显示存在HER2高水平扩增（拷贝倍数为20），同时伴有EGFR L858R突变。在患者家属知情同意后，尝试给予患者德曲妥珠单抗治疗。考虑到患者PS较差（ECOG PS 2分），采用减量方案（4.4 mg/kg，每3周给药1次）。作为四线治疗方案，首剂给药时间为2023年12月1日。2024年2月22日评估疗效达PR（RECIST 1.1 版）。治疗后肺部、纵隔3A组淋巴结及肾上腺病灶（图2）显著缩小，血清肿瘤标志物水平亦明显下降。此外，患者总体耐受性良好，依据不良事件通用标准术语（Common Terminology Criteria for Adverse Events, CTCAE）5.0版，不良反应仅包括轻度头晕（1级）、乏力（1级）、吞咽困难（食管钡餐造影未见异常，1级）。2024年4月9日，患者小脑出现新发转移灶，评估为疾病进展（progressive disease, PD），此时肺部、3A组淋巴结及肾上腺病灶大小仍保持稳定，ECOG PS仍为2分。2024年5月24日血浆NGS检测未检测到HER2扩增。后续治疗中予尝试使用伏美替尼，但患者很快出现严重肝损伤，PS急剧恶化，最终于2024年7月13日因肿瘤相关原因死亡。

**图1:**
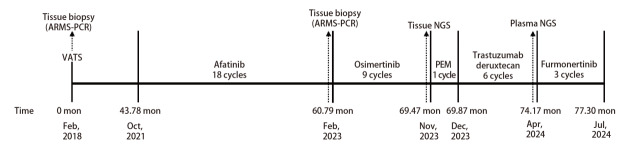
患者自确诊到死亡的事件时间轴

**图2:**
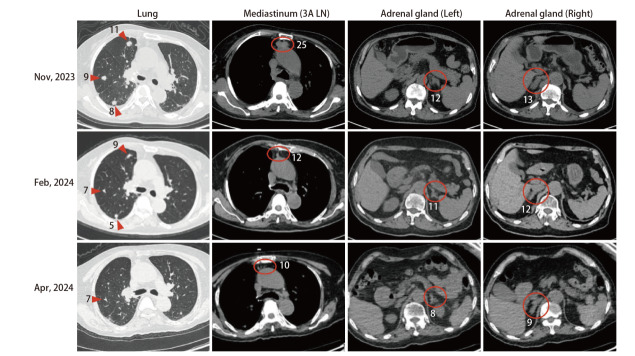
德曲妥珠单抗治疗前（2023年11月）、疗效评估为PR时（2024年2月）及疾病进展时（2024年4月）的肺部、血管前（3A组）淋巴结及肾上腺连续高分辨率 CT影像对比（疗效评估标准：RECIST 1.1版）。图中标注数字为病灶测量最长径（单位：mm）。

## 2 讨论

本病例报道了后线德曲妥珠单抗对EGFR-TKIs治疗后出现获得性HER2扩增的转移性肺腺癌具有潜在疗效。本例患者为老年女性，肺腺癌根治术后数年出现疾病复发，一线姑息治疗为针对转移性肺腺癌的分子靶向治疗。在二线奥希替尼治疗进展后，经NGS检出HER2高水平扩增，根据患者情况，予个体化德曲妥珠单抗减量使用，2个月后疗效评估为PR。经4个月治疗后，患者因小脑新发转移灶判定为PD，而其余多处病灶仍保持稳定。后续血浆NGS结果进一步证实，HER2扩增被抑制。

HER2由HER2/ERBB2原癌基因编码，其表达上调可参与肿瘤发生及进展^[9]^。HER2异常改变类型包括基因突变、基因扩增及蛋白过表达^[4]^。II期DESTINY-Lung01^[2]^和DESTINY-Lung02^[3]^研究已证实，德曲妥珠单抗用于经治HER2突变型NSCLC疗效显著且安全性可控。2025年世界肺癌大会上发布了DESTINY-Lung05研究^[10]^的最终结果，中位随访时间延长至20个月以上，观察到德曲妥珠单抗治疗既往经治HER2突变晚期NSCLC患者的中位无进展生存期（progression-free survival, PFS）达9.9个月，中位总生存期（overall survival, OS）达21.0个月，证实了德曲妥珠单抗在中国HER2突变晚期NSCLC患者后线治疗中的疗效，为其在临床实践中的应用提供重要依据。此外，目前多项研究正在优化其联合方案与脑转移人群的治疗策略。然而，该ADC在HER2扩增患者中的疗效数据仍十分有限。目前，德曲妥珠单抗已获批用于不可切除或转移性HER2突变型NSCLC的二线治疗，但尚未获批用于HER2扩增型NSCLC^[11]^。作为少见分子亚型，HER2扩增在NSCLC患者中占比仅2%-5%^[4]^。

本病例显示，在通过转移淋巴结组织NGS检出HER2扩增后，予以德曲妥珠单抗治疗可达到PR。既往数据中，DESTINY-Lung01研究^[2]^中有2例患者同时存在HER2突变与扩增，均观察到显著疗效。此外，Yun等^[12]^报道了1例NSCLC患者在接受帕博利珠单抗联合化疗（卡铂联合培美曲塞）进展后，采用德曲妥珠单抗联合靶向化疗作为二线方案，患者获得深度缓解，缓解持续时间达到13.8个月。

值得注意的是，本病例中HER2扩增可能是奥希替尼获得性耐药的机制之一。HER2扩增可作为奥希替尼原发性或获得性耐药的机制^[8]^。但迄今为止，尚无队列研究证实ADC类药物对此类奥希替尼获得性耐药患者的疗效。本病例中，患者多处转移灶对德曲妥珠单抗产生显著且持久的疗效，提示ADC类药物在EGFR-TKIs获得性耐药中具有潜在应用价值。此外，德曲妥珠单抗可能同样适用于靶向治疗后出现获得性HER2扩增的其他病例。Vakkalagadda等^[13]^报道了1例伴有获得性HER2扩增的RET融合NSCLC患者，采用德曲妥珠单抗联合塞尔帕替尼治疗后安全有效。本例在德曲妥珠单抗治疗后出现小脑新发转移灶，可能与原发肿瘤与转移肿瘤HER2表达异质性有关；此外，德曲妥珠单抗作为大分子ADC，影响血脑屏障通透性和脑局部药物浓度；作为四线治疗，颅内微转移或在该药治疗前就已存在。血浆NGS未检出HER2扩增但仍发生进展的可能原因，包括液体活检局限性（假阴性、组织存在扩增但仍未释放到血液）、发生旁路耐药（MET扩增、EGFR旁路、PIK3CA突变）等。

既往研究^[2,3,10]^表明，德曲妥珠单抗使用的安全性可控。本例患者使用德曲妥珠单抗后发生的不良反应包括头晕、乏力和吞咽困难，CTCAE均为1级，经对症治疗或观察后可好转，表明该患者对于德曲妥珠单抗治疗的耐受性可。

对于扩增型NSCLC，应谨慎选择单用ADC或ADC联合TKIs治疗方案。鉴于本例患者PS较差，我们采用了减量ADC单药治疗。而进展后血浆NGS仅检出EGFR L858R突变，未检测到HER2扩增，提示进展可能与EGFR突变有关。减量应用仍获得持久缓解，也提示个体化剂量选择对PS不佳人群的可行性。此外，HER2扩增水平同样是需要考量的因素。近期一项研究^[14]^显示，0.9%的肺腺癌存在HER2高水平扩增（≥6拷贝），且ADC对此类人群有效。本病例也表明，对于肿瘤患者治疗随访过程中，需进行动态、全面的分子监测。本文存在一些不足之处。首先，报道的病例既往接受过分子靶向、化疗、放疗等多种治疗，可能影响最终疗效的判断。此外，作为单个病例，德曲妥珠单抗疗效或受其他因素综合影响，其应用仍需大样本临床确证性研究证实。
